# Chemical Composition and Transgenerational Effects on *Caenorhabditis elegans* of Seasonal Fine Particulate Matter

**DOI:** 10.3390/toxics11020116

**Published:** 2023-01-24

**Authors:** Rongying Yang, Pengxiang Ge, Xiaoming Liu, Wankang Chen, Zhansheng Yan, Mindong Chen

**Affiliations:** Collaborative Innovation Center of Atmospheric Environment and Equipment Technology, Jiangsu Key Laboratory of Atmospheric Environment Monitoring and Pollution Control, School of Environmental Science and Engineering, Nanjing University of Information Science & Technology, Nanjing 210044, China

**Keywords:** PM_2.5_, PM_1_, chemical composition, *Caenorhabditis elegans*, offspring, apoptosis

## Abstract

While numerous studies have demonstrated the adverse effects of fine particulate matter (PM) on human health, little attention has been paid to its impact on offspring health. The multigenerational toxic effects on *Caenorhabditis elegans* (*C. elegans*) were investigated by acute exposure. PM_2.5_ and PM_1_ samples were collected and analysed for their chemical composition (inorganic ions, metals, OM, PAHs) in different seasons from April 2019 to January 2020 in Lin’an, China. A higher proportion of organic carbon components (34.3%, 35.9%) and PAHs (0.0144%, 0.0200%) occupied the PM_2.5_ and PM_1_ samples in winter, respectively. PM_1_ in summer was enriched with some metal elements (2.7%). Exposure to fine PM caused developmental slowing and increased germ cell apoptosis, as well as inducing intestinal autofluorescence and reactive oxygen species (ROS) production. PM_1_ caused stronger toxic effects than PM_2.5_. The correlation between PM component and F0 generation toxicity index was analysed. Body length, germ cell apoptosis and intestinal autofluorescence were all highly correlated with Cu, As, Pb, OC and PAHs, most strongly with PAHs. The highest correlation coefficients between ROS and each component are SO_4_^2−^ (R = 0.743), Cd (R = 0.816) and OC (R = 0.716). The results imply that OC, PAHs and some transition metals play an important role in the toxicity of fine PM to *C. elegans*, where the organic fraction may be the key toxicogenic component. The multigenerational studies show that PM toxicity can be passed from parent to offspring, and gradually returns to control levels in the F3–F4 generation with germ cell apoptosis being restored in the F4 generation. Therefore, the adverse effects of PM on reproductive damage are more profound.

## 1. Introduction

As the country’s economy expands in China, air pollution has become an increasingly serious problem, with particulate matter pollution being the most important issue. Epidemiological studies have shown that particulate matter (PM) is closely related to the morbidity and mortality of human diseases [1]. Studies have shown that the smaller the particle size, the slower the settling rate, resulting in longer retention time in the air, deeper entry into the respiratory tract and, therefore, greater impact on human health [2]. PM_2.5_ (aerodynamic diameter d ≤ 2.5 µm) and PM_1_ (aerodynamic diameter d ≤ 1 µm) are risk factors for stroke, cardiovascular diseases (CVD) and respiratory diseases, with PM_1_ accounting for the majority of mortality due to PM_2.5_. Study has shown that PM of smaller particle size has a greater impact on mortality [3]. Numerous epidemiological and experimental studies have shown that long-term PM_2.5_ and PM_1_ exposure is positively associated with elevated blood pressure and hypertension prevalence. The effect of PM_2.5_ on hypertension is likely to be primarily caused by PM_1_ [4]. Long-term PM_1_ exposure has a higher negative impact on CVD and childhood pneumonia compared to PM_2.5_ [5,6]. There is a stronger correlation between mortality from chronic obstructive pulmonary disease and smaller PM, with the main toxic component coming from PM less than 0.3 µm in diameter [7]. In addition, increased incidence of type 2 diabetes and higher fasting blood glucose levels are associated with exposure to high levels of PM_2.5_, PM_1_ and NO_2_ [8].

There are differences in the substances adsorbed by different particle sizes, and their chemical composition is a key factor in the different toxic effects [9]. Cytotoxicity from PM_2.5_ exposure is revealed to be significantly correlated with heavy metals and organic components, but less so with water-soluble ions [10]. SO_4_^2−^, NO_3_^−^, Ca^2+^, NH_4_^+^ and water-soluble organic carbon (WSOC) dominate the water-soluble fractions in order of predominance, while metals are less predominant and show significant spatial and seasonal variation [11,12]. Heavy metal components in PM, such as As, Ni and Pb, have adverse effects on human health and risk of cancer, among which As is the highest risk for children [13,14]. Carbon components (OC, EC) and secondary inorganic aerosols (SOA) in the chemical composition of PM_2.5_ and PM_1_ are most associated with cardiovascular disease mortality, with OC and EC showing relatively high concentrations in winter compared to summer [15]. Polycyclic aromatic hydrocarbons (PAHs) contained in PM are highly carcinogenic and play a significant role in oxidative stress, DNA damage and gene mutation [16,17,18]. Among PAHs, Benzo(a)anthracene (BaA), Benzo(a)pyrene (BaP), Benzo(b)fluoranthene (BbF) and Dibenzo-(a,h)anthracene (DaA) are potentially carcinogenic, with higher cancer risks from BaA, BaP and BbF in winter than in summer, and from DaA in summer than in winter [14]. Organic components of PM may cause a risk of CVD and damage to the cardiovascular system [19]. Primary organic aerosols (POA) were significantly correlated with ROS, LDH and IL-6, suggesting that organic compounds play a key role in the production of cellular inflammatory and oxidative responses [20].

The whole genome sequence of *Caenorhabditis elegans* (*C. elegans*) is known and has 60–80% homology with human genes [21]. It is an excellent model animal for environmental toxicology research, and its health effects and toxicogenic mechanisms have important implications for humans and other higher mammals [21,22]. It has been widely used to evaluate the toxicity of environmental pollutants such as soil, endocrine disruptors, heavy metals and nanomaterials [23,24,25,26]. The impact of *C. elegans* on growth and development, the nervous system and gene expression was assessed by measuring various indicators such as body length, ROS levels and germ cell apoptosis. Studies have demonstrated that long-term exposure to traffic-related PM_2.5_ and coal-combustion-related PM_2.5_ adversely affects *C. elegans* longevity, development, reproduction, locomotor behaviour and gut function. Oxidative stress and abnormal defecation behaviour may be key factors in the adverse effects of coal-combustion-associated PM_2.5_ on *C. elegans*, and studies have shown that long-term exposure has more severe adverse effects on *C. elegans* than acute exposure [27,28]. Previous studies have confirmed that exposure not only causes a significant reduction in the length, brood size and lifespan of parental *C. elegans*, but also has a negative impact on the next generation [29,30,31]. This indicates that *C. elegans* is widely used in toxicological studies, especially in the study of transgenerational effects. The proportion of various chemical components in fine PM is affected by season and particle size, resulting in spatial and temporal differences in their impact on human health [32]. Since no specific conclusions have been reached on the key toxic components of fine particulate matter, and there are few studies on the health effects of different particle sizes on the offspring of fine PM, in this study, we collected PM_2.5_ and PM_1_ samples from the Lin’an area over four seasons to search for determinants in fractions by analysing the toxic effects, and investigating the effects of PM with different particle sizes on the multigenerational toxicity of *C. elegans*.

## 2. Materials and Methods

### 2.1. Particulate Matter Collection

The PM_2.5_ and PM_1_ samples were collected at the Lin’an Atmospheric Background Station (30°18′ N, 119°45′ E) from 1 April 2019 to 31 January 2020, with January–February, April–May, July–August and September–October representing the four seasons of winter, spring, summer and autumn, respectively. There were 61 samples in spring, 62 samples in summer, 61 samples in autumn and 62 samples in winter for particulate matter collection. The station is located about 50 km from Hangzhou, 210 km from Shanghai and 250 km from Nanjing, and is surrounded by forest vegetation, which is less affected by man-made pollution. Two medium-flow samplers (KB-120F, SunGard, Qingdao, China), respectively equipped with PM_2.5_ and PM_1_ cutting heads, were used to collect particulate matter, with a sampling flow rate of 100 L/min and a sampling time of 22 h per day. The whole particulate matter was extracted by ultrasonication in an ice water bath. The filter membrane was cut and added to ultrapure water to sonicate twice for 30 min. The extract was filtered through gauze, then placed in a freeze dryer for one week to obtain a particulate matter sample [33].

### 2.2. Chemical Composition of PM_2.5_ and PM_1_

The seasonal samples were analysed for anions (F^−^, Cl^−^, SO_4_^2−^, NO_3_^−^) and cations (Na^+^, NH_4_^+^, K^+^, Mg^2+^, Ca^2+^). Water-soluble inorganic ion samples were obtained by placing the particles in a centrifuge tube and adding ultrapure water, then sonicating them in an ice-water bath and filtering them through a 0.22 µm filter head. Anions were detected by ion chromatography (ICS-2000, Dionex, Sunnyvale, CA, USA) using 10 mM KOH as eluent at 1 mL/min. Cations were detected by ion chromatography (ICS-3000, Dionex, Sunnyvale, CA, USA) using 10 mM methanesulfonic acid as eluent at 1 mL/min. The inductively coupled plasma mass spectrometer (ICP-MS, Thermo Fisher Scientific, Waltham, MA, USA) was used to detect heavy metal elements. The samples were prepared using a microwave digester (GEM Corporation, Charlotte, NC, USA). A certain mass of PM was weighed into the ablation tube and 5 mL of HNO_3_ (GR) was added; then, the ablated samples were filtered and sealed for testing. Al, Fe, Zn, As, Ba, Cu, Cr, Cd, Co, Pb, Mn, Sb, Sr, Se, Ni, V and Ti in the sample were then measured.

PAHs were detected by Gas Chromatograph–Mass Spectrometer (GC-MS, NYSE: A, Palo Alto, CA, USA). A sufficient mass of PM was weighed and dissolved in CH_2_Cl_2_, and 20 µL of standard solution containing isotope markers was added to each sample. The samples were sonicated to fully dissolve, then filtered and nitrogen blown to 200 µL for testing. The samples were injected in a volume of 2 µL using a split-flow injection and separated using a capillary column HP-5 MS (30 m × 250 µm × 0.25 µm). Organic carbon (OC) and element carbon (EC) in PM samples were measured using a thermal/optical carbon analyser (Sunset model 4, Sunset Laboratory Inc, Forest Grove, OR, USA), which performs OC and EC measurements using thermal and optical (TOT) methods. The instrument provides eight components at the end of the measurement, defining OC and EC as follows:

OC = OC_1_ + OC_2_ + OC_3_ + OC_4_ + OPC (1)

EC = EC_1_ + EC_2_ + EC_3_-OPC (2)

The carbon mass analyser was calibrated daily with CH_4_ and repeated at a rate of once every 10 samples. OC and EC errors were less than 10% and detection limits were both less than 1 μg/m^3^ [34]. Details can be found in the supporting information.

### 2.3. C. elegans Strains and Maintenance

The *C. elegans* strain used in this study was the wild-type N_2_ Bristol strain, provided by the Caenorhabditis Genetics Center (CGC). They were maintained on nematode growth medium at 20 °C, and inoculated with *Escherichia coli* OP50 as a food source [35].

### 2.4. Experimental Design

**Figure 1:**
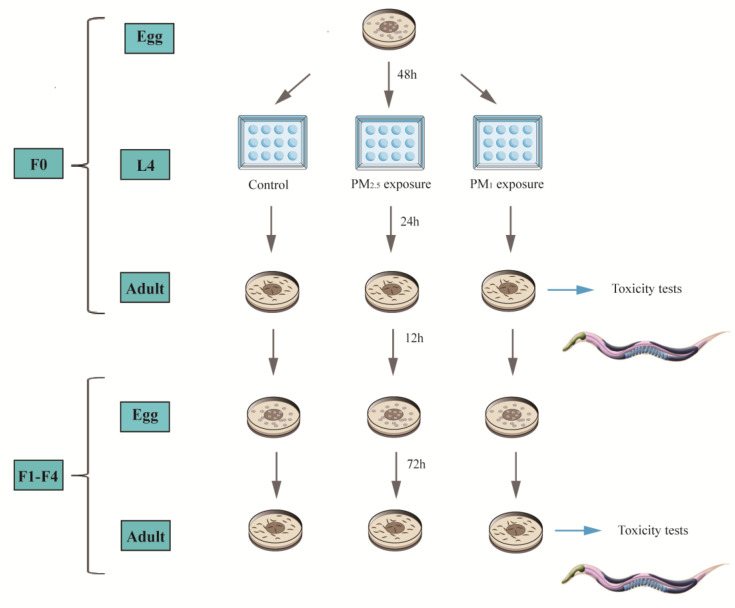
Schematic diagram of the acute exposure experiment using *C. elegans*.

The PM was suspended and diluted using K-medium. The following 5 concentration gradients were set up: 0 (control), 1 mg/L, 10 mg/L, 100 mg/L, 1000 mg/L. Acute maternal exposure was used to investigate the effects of particulate matter on the health of offspring. Figure 1 shows the acute exposure experiment of *C. elegans* during the experiment. We selected gravid nematodes, rinsed them from the plates into centrifuge tubes and lysed for 2–3 min by adding K-medium and Clorox solution successively at 1:1 [36]. The lysed eggs from the bottom of the centrifuge tubes were transferred to NGM plates with OP50 and incubated in a biochemical incubator at 20 °C for 48 h to obtain the L4 larvae, which were finally transferred to 12-well plates for 24 h of exposure. A total of 2 mL of exposure solution was added to each well and 100 µL of OP50 was added as a food source to ensure unrestricted nematode growth. L4 larvae were incubated at 20 °C for 24 h to obtain F0 adults, and the toxicity index of F0 adults was determined. F1 eggs were obtained by synchronising F0 gravid nematodes and cultured for 72 h to obtain F1 adults, and then F1 adults were assayed for toxicity indicators. The above process was continued to complete the index determination for multiple generations (F0~F4).

### 2.5. Toxicity Indicators Tests

#### 2.5.1. Body Length Assay

The exposed nematodes were collected and then placed on slides. After cauterisation with an alcohol lamp, photographs were taken under the microscope using ImageView 9.0 software; the images were processed by ImageJ V1.8.0 software to calculate body length. At least 30 nematodes were selected from each treatment group for measurement, and this was repeated three times.

#### 2.5.2. Intestinal Autofluorescence Assay

Intestinal autofluorescence caused by lysosomal deposits of lipofuscin can accumulate over time in aging nematodes or nematodes exposed to specific toxicants, and the analytical method was performed as described previously [37,38]. The exposed nematodes were anaesthetised with levamisole solution (60 µM), and then pipetted onto a slide containing a 2% agarose pad. Fluorescence intensity images of each group of nematodes intestinal autofluorescence were taken under a fluorescence microscope (UV-2A filter). The images were analysed by ImageJ V1.8.0 software and the results were expressed as mean fluorescence intensity values. A minimum of 30 nematodes were detected in each group, and the process was repeated three times.

#### 2.5.3. ROS Assay

ROS reflect levels of oxidative stress and are an important contributor to aging and disease [39]. ROS production in nematodes was measured using the 5′,6′-chloromethyl-2′,7′-dichlorodihydrofluorescein diacetate (CM-H_2_DCFDA) fluorescent probe method [40]. The exposed nematodes were collected after washing 3 times with K-medium, and then placed in NGM plates containing 1 mL of CM-H2DCFDA (50 µM), fed OP50 and incubated for 2 h in an incubator at 20 °C. After staining, the nematodes were washed 3 times with K-medium. They were anaesthetized with levamisole solution (60 µM), and then pipetted onto a slide containing a 2% agarose pad. The fluorescence intensity of each group of nematodes was observed under a fluorescent microscope (FITC filter). The images were captured using ImageView 9.0 software and were analysed by ImageJ V1.8.0 software. A minimum of 30 nematodes were tested in each group and the process repeated three times.

#### 2.5.4. Apoptosis Assay

Apoptotic germ cells were counted by acridine orange (AO) in vivo staining [41]. After exposure, nematodes were transferred to NGM plates with OP50, to which l mL of acridine orange solution (25 µg/mL) was added, and were incubated at 20 °C for 60 min. After staining, the nematodes were transferred to NGM with OP50 and recovered in the dark for 40 min. The stained nematodes were anesthetized with levamisole solution (60 µM) after 3 washes, then transferred to slides containing w = 2% agarose pads. The slides were placed under a fluorescent microscope (FITC filter) for observation and photographed. AO can cause apoptotic cells to stain with yellow-green debris particles, which are brighter than normal cells. At least 30 nematodes were tested in each group and the process replicated three times.

### 2.6. Statistical Analysis

The experimental results were expressed as means ± standard error of the mean (SEM). Data analysis and charting were carried out using SPSS 24.0 (SPSS Inc., Chicago, IL, USA) and Origin 2018 (OriginLab, Northampton, MA, USA) software, respectively. The key toxicogenic factors in chemical fractions were analysed using the experimental approach of factorial analysis, where the larger the Pearson correlation coefficient, the higher the correlation. The correlation between two variables was considered significant if *p* < 0.05 and statistically significant if *p* < 0.01. Statistical analysis of transgenerational effects on *C. elegans* of seasonal fine particulate matter was carried out, where one-way ANOVA was used for comparisons between multiple groups and Dunnett’s test was used for comparisons between control and exposed groups. The difference between the two groups was considered significant if *p* < 0.05 and statistically significant if *p* < 0.01.

## 3. Results and Discussion

### 3.1. PM_2.5_ and PM_1_ Concentrations

The changing trends of PM_2.5_ and PM_1_ mass concentrations in the Lin’an area were essentially the same throughout the year, showing the trend: winter > spring > autumn > summer. The highest point of the year was owing to the frequent occurrence of temperature inversion conditions in winter, which made it difficult for air pollution to spread [42]. PM_1_ accounted for 74.2–80.6% of the mass concentration of PM_2.5_ (Table S1), and was the main component of PM_2.5_. The result demonstrated the importance of submicron particles.

### 3.2. PM Chemical Composition Analysis

#### 3.2.1. Water-Soluble Inorganic Ions

**Figure 2:**
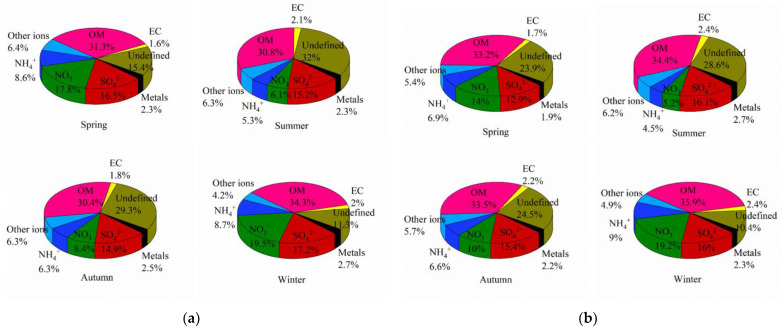
Percentage of each component in PM collected for four seasons: (**a**) PM_2.5_; (**b**) PM_1_.

Water-soluble ions contributed 49.4%, 32.9%, 35.9% and 49.6% of PM_2.5_ mass, and 39.3%, 32.0%, 37.7% and 49.1% of PM_1_ mass in each of the four seasons (Figure 2). The high agreement between the mass share of water-soluble ions and the change in PM concentration indicated that water-soluble ions were an important component of PM. The proportion of water-soluble ions in PM_2.5_ and PM_1_ followed the same trend with distinct seasonal variation characteristics, generally lower in summer than in winter, as confirmed by previous studies [43,44]. This may be due to the fact that the prevailing maritime monsoon and frequent precipitation in summer largely reduced atmospheric pollutants. As a result, the summer months accounted for a lower mass of water-soluble ions throughout the year. SO_4_^2−^, NO_3_^−^ and NH_4_^+^ (SNA) were its main inorganic ions, formed by the atmospheric transformation of precursor gases (NH_3_, SO_2_ and NO_x_) and represent secondary pollution [45]. The proportion of SNA in the four seasons of Lin’an was 80.6–91.4% of the total water-soluble inorganic ions. The seasonal variation characteristics of different ions were not entirely consistent due to different pollution sources, meteorological factors, etc. Seasonal trends showed that SNA accounted for a higher proportion in winter, which can be attributed to higher precursor concentrations and unfavourable atmospheric dispersion conditions in that season. It was noteworthy that the NO_3_^−^ proportions were both highest in winter (19.45%, 19.19%) and lowest in summer (6.13%, 5.22%), with significant seasonal differences, due to the fact that low temperatures helped gaseous nitric acid to form particulate aerosols such as ammonium nitrate, while high temperatures helped the decomposition of nitrate. Therefore, the main reason for the difference between winter and summer was that NO_3_^−^ changes were influenced by a combination of air mass transport and temperature, making the proportion of NO_3_^−^ much higher in winter than in summer [46]. The NO_3_^−^/ SO_4_^2−^ ratio can be used to determine whether the pollution comes mainly from stationary sources (coal combustion) or mobile sources (motor vehicle exhaust), with a ratio greater than 1 indicating that mobile sources were the main contributor, and less than 1 indicating that stationary sources contribute more [47]. The NO_3_^−^/ SO_4_^2−^ ratios for PM_2.5_ and PM_1_ were essentially the same for all four seasons (Table S2), indicating that the pollution sources of PM_2.5_ and PM_1_ in Lin’an were consistent. The ratios indicated that the site was dominated by stationary sources in summer and autumn and mobile sources in spring and winter.

#### 3.2.2. Heavy Metal Elements

The sum of all analysed elements explained approximately 1.9–2.7% of the PM sample mass (Figure 2). Fe, Al and Zn were the most abundant, which accounted for more than 90% of the total mass of elements analysed. Other trace elements such as Ba, Bi, Cd, Co, Cr and Cs were present at low levels, accounting for about 5.3–6.2% of the total amount analysed. Of these, Fe and Al—as crustal elements—were mainly derived from soil and dust, while the presence of high proportions of Zn, Cu, Pb and Mn detected in PM_2.5_ and PM_1_ samples may be directly related to vehicle emissions due to the proximity of sampling points to high-traffic-flow paths [48]. Cu and Pb mainly originated from motor vehicle emissions and industrial emissions, and both showed similar trends in the proportion of PM_2.5_ and PM_1_ samples. They both accounted for the highest proportion in winter, indicating that traffic emissions were the main source of Cu and Pb in winter, which was consistent with the trend of NO_3_^−^ contribution. The PM_2.5_ samples had a higher proportion of total elements analysed in winter, while the highest in the PM_1_ samples was in summer (Table S3). Overall, the total contribution of the metal fractions was small and did not vary significantly seasonally.

#### 3.2.3. PAHs

PAHs accounted for 0.0101%, 0.0036%, 0.0079% and 0.0142% of the sample’s mass and 0.0140%, 0.0041%, 0.0121% and 0.0200% of the PM_1_, respectively (Table 1). The mass contribution of PAHs measured in the samples was high in spring and winter with 0.0101% and 0.0142% for PM_2.5_ and 0.0140% and 0.0200% for PM_1_, respectively, and lowest in summer with 0.0041% for PM_1_ and 0.0036% for PM_2.5_. The largest contributions occurred in winter, when low temperatures and weak atmospheric radiation made PAHs less susceptible to photodegradation and facilitated the accumulation of PAHs on the surface of particles. The lowest contributions were found in summer, mainly due to the high temperature, the intensity of solar radiation and low atmospheric stability, which made PAHs susceptible to photodegradation. The PAH contributions of PM_2.5_ and PM_1_ showed the same seasonal trend: winter > spring > autumn > summer. This was consistent with the results of most previous studies, indicating that organic compounds in PM were generally lower in summer than in winter [49]. Meanwhile, the PAH contributions of PM_1_ in four seasons were higher than those in PM_2.5_ samples. Most previous studies had found that PAHs were mainly found in fine particles, which may account for the differences in PAH concentrations in different particle sizes [50].

#### 3.2.4. OC/EC

Generally, Organic matter (OM) was the important component of PM, and an OM/OC mass ratio of 1.6 was used in atmospheric aerosols. OM contributions were above 30% of PM_2.5_ and PM_1_ mass in four seasons. The percentage of OM in PM_2.5_ samples was significantly highest in winter (34.9%), while the percentage of OM in PM_1_ samples was slightly higher in winter (36.0%) compared to the rest of the seasons (Figure 2). This suggested that there were more combustion sources in winter, whereas high temperatures in summer resulted in semi-volatile organic compounds being present mainly in gaseous form, hence the lower OC contribution in summer. In addition, the lower EC concentrations indicated that there were no significant sources of incomplete combustion in the vicinity of the sampling sites. The OC/EC ratio was often used to identify the secondary sources of PM, and the OC/EC ratio in Lin’an was mainly distributed between 4 and 16. Based on previous studies, it was suggested that vehicle exhaust and coal combustion were the main sources of the carbon fraction in fine particles in the region [51]. Except for the EC, which showed no significant seasonal differences, the TC, OC and secondary organic aerosol (SOC) in the PM_2.5_ and PM_1_ showed the trend of highest in winter and lowest in summer (Table S4). This was consistent with previous studies that SOC tended to form more often in dry and cold winters [52]. This was mainly due to meteorological conditions in winter which were not conducive to OC diffusion. Meanwhile, due to the high level of coal-fired heating and motor vehicle use in winter, a large amount of motor vehicle exhaust combined with particulate matter, resulting in high SOC. The contribution of OC to PM_1_ was higher than that to PM_2.5_, mainly because the smaller the particle size, the higher the organic matter adsorbed on the PM or secondary production. The unmeasured fraction was higher in summer and lower in winter, probably due to greater sample loss during the dry and hot seasons.

### 3.3. Toxicity of Seasonal PM_2.5_ and PM_1_ in C. elegans

#### 3.3.1. Body Length

**Figure 3:**
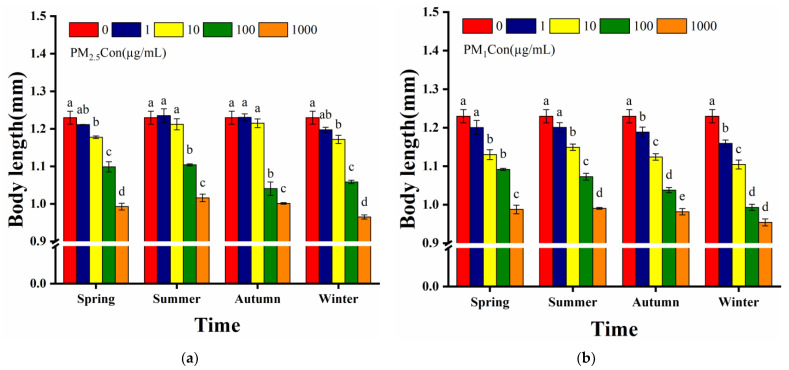
Effect of different seasons of PM exposure on nematode body length: (**a**) body length of nematodes exposed to 1, 10, 100 and 1000 µg/mL of PM_2.5_ in four seasons compared to the control; (**b**) body length of nematodes exposed to 1, 10, 100 and 1000 µg/mL of PM_1_ in four seasons compared to the control. L4 stage larvae were selected for 24 h acute exposure. Different letters (a, b, c, d, e) indicate significant differences among the five PM treatments in each season (*p* < 0.05). Bars represent means ± SEM.

To investigate the adverse effects of PM on the growth and development of nematodes, the body length of post-exposure nematodes was analysed. We observed that acute exposure to low concentrations (1–10 µg/mL) of PM_2.5_ in summer and autumn did not significantly shorten the body length of nematodes, and body length was significantly reduced (*p* < 0.001) by exposure to high concentrations of PM_2.5_ (100–1000 µg/mL) in all four seasons (Figure 3a). In contrast, acute exposure to PM_1_ only in spring and summer at low exposure concentrations (1 µg/mL) did not significantly shorten nematode body length, while exposure to the remaining PM_1_ samples at 10–1000 µg/mL significantly reduced the body length (*p* < 0.001) (Figure 3b). The dependence of the toxic effect increased with increasing doses of PM_2.5_ and PM_1_ concentrations, and exposure to high concentrations of the toxic solution caused significant reduction in the body length of nematodes. The toxicity of PM_2.5_ and PM_1_ in four seasons varied significantly, with acute exposure to PM_2.5_ and PM_1_ in winter greatly shortening body length of nematodes and showing the highest toxicity and the lowest toxicity in summer. The body length of the nematodes exposed to high concentrations of PM_1_ was significantly shorter than that of PM_2.5_ in the same season, suggesting that PM_1_ inhibited the growth and development of nematodes more significantly than PM_2.5_.

#### 3.3.2. Intensity of Intestinal Autofluorescence

**Figure 4:**
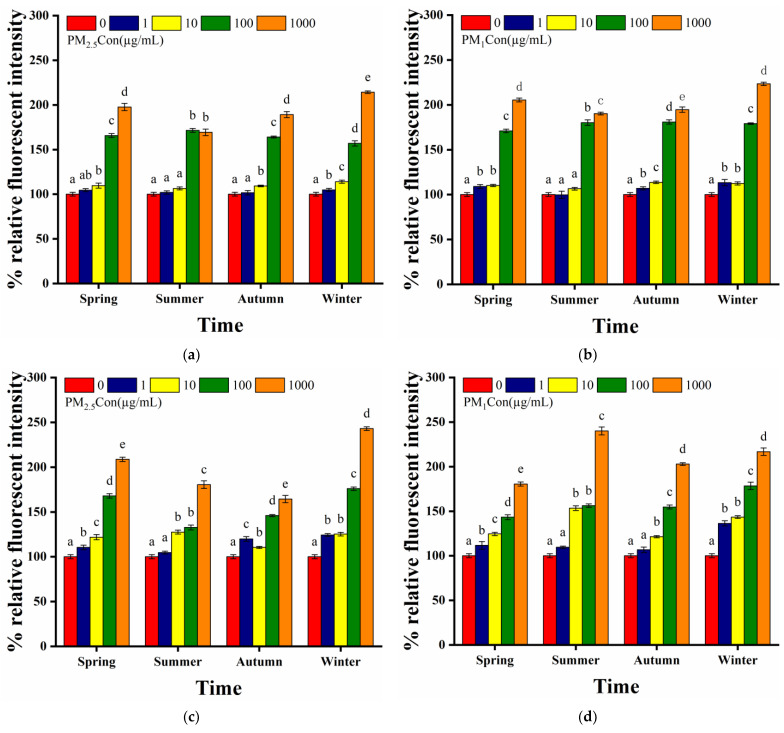
Effect of different seasons of PM exposure on nematode intestinal autofluorescence and ROS levels: (**a**) intestinal autofluorescence levels of nematodes exposed to 1, 10, 100 and 1000 µg/mL of PM_2.5_ in four seasons compared to the control; (**b**) intestinal autofluorescence levels of nematodes exposed to 1, 10, 100 and 1000 µg/mL of PM_1_ in four seasons compared to the control; (**c**) ROS levels of nematodes exposed to 1, 10, 100 and 1000 µg/mL of PM_2.5_ in four seasons compared to the control; (**d**) ROS levels of nematodes exposed to 1, 10, 100 and 1000 µg/mL of PM_1_ in four seasons compared to the control. L4 stage larvae were selected for 24 h acute exposure. Different letters (a, b, c, d, e) indicate significant differences among the five PM treatments in each season (*p* < 0.05). Bars represent means ± SEM.

Toxic substances usually caused damage to the intestinal and reproductive functions of nematodes [53]. Intestinal autofluorescence in nematodes was produced by lipofuscin accumulation induced by tissue senescence, and represents the degree of senescence in nematodes. The effect of PM on nematodes’ gut development was studied by the intensity of the gut’s intestinal fluorescence. Acute exposure to PM_2.5_ in all four seasons at low concentrations (1 µg/mL) did not cause a significant increase in the intensity of intestinal autofluorescence, indicating that the toxic effect of low concentrations of PM_2.5_ was not significant. Meanwhile, exposure to PM_2.5_ at high concentrations (100–1000 µg/mL) caused a significant increase in the intensity of intestinal fluorescence (Figure 4a). Thus, exposure to high concentrations of PM_2.5_ caused significantly higher intestinal fluorescence intensity than low concentrations. We observed that acute exposure to PM_1_ in summer at low concentrations (1–10 µg/mL) did not cause a significant increase in the level of intestinal autofluorescence. However, acute exposure to PM_1_ in winter at low concentrations (1–10 µg/mL) caused a significant increase in intestinal autofluorescence (*p* < 0.01). Exposure to high concentrations of PM_1_ (100–1000 µg/mL) in all four seasons induced a highly significant increase in intestinal autofluorescence (*p* < 0.001) (Figure 4b). In terms of seasonal differences, we observed that winter samples induced much greater intestinal fluorescence intensity than summer, with little difference between spring and autumn, suggesting a greater degree of damage to intestinal development from PM in winter. Compared to PM_2.5_, exposure to high concentrations of samples in the same season resulted in higher intestinal fluorescence intensity when induced by PM_1_, to which nematodes were more sensitive.

#### 3.3.3. ROS Production

Oxidative stress was an important indicator for evaluating PM toxicity. We observed that acute exposure to only the lowest concentration of PM_2.5_ (1 µg/mL) in summer did not result in a significant increase in ROS production in the nematode gut, except for exposure to the rest of the concentrations (10–1000 µg/mL) in summer and all other seasonal concentrations which induced a significant increase in ROS production (*p* < 0.05) (Figure 4c). Among the four seasons, exposure to PM_2.5_ in winter induced the highest ROS production, which was consistent with previous findings that PM_2.5_ in winter was the most impaired in terms of antioxidant capacity [54]. After acute exposure, ROS production in nematodes did not increase significantly only at the lowest concentration in the autumn PM_1_ samples; for the rest of the concentrations, a significant increase in ROS was induced. Additionally, ROS in nematodes induced by PM_1_ samples were higher in winter and summer among the four seasons, with the highest ROS induced in summer (Figure 4d). ROS production was significantly higher at acute exposure of 1000 µg/mL summer PM_1_ than PM_2.5_, while acute exposure of 1000 µg/mL of winter PM_1_ resulted in lower ROS levels than that of winter PM_2.5_, with the highest ROS production at 1000 µg/mL of PM_1_ in summer.

#### 3.3.4. Number of Germ Cells Apoptosis

**Figure 5:**
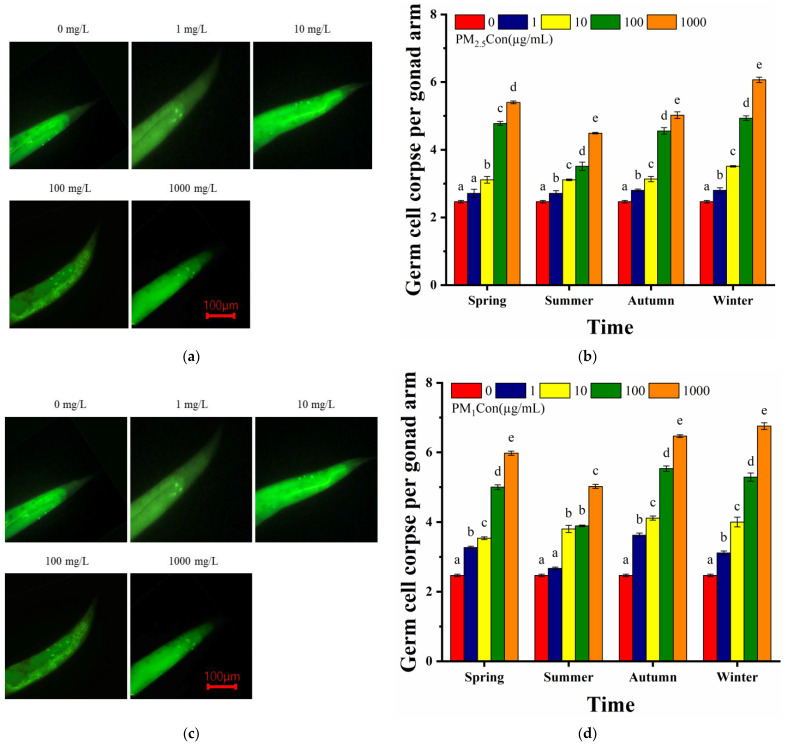
Effect of different seasons of PM exposure on nematodes germ cell apoptosis: (**a**) the plot of germ cell apoptosis at different PM_2.5_ concentrations; (**b**) germ cell apoptosis of nematodes exposed to 1, 10, 100 and 1000 µg/mL of PM_2.5_ in four seasons compared to the control; (**c**) the plot of germ cell apoptosis at different PM_1_ concentrations; (**d**) germ cell apoptosis of nematodes exposed to 1, 10, 100 and 1000 µg/mL of PM_1_ in four seasons compared to the control. L4 stage larvae were selected for 24 h acute exposure. Different letters (a, b, c, d, e) indicate significant differences among the five PM treatments in each season (*p* < 0.05). Bars represent means ± SEM.

Reproductive toxicity was a sensitive and important endpoint for studying the transgenerational effects of other toxicants [29]. We observed significant differences in the number of apoptotic germ cells between treatment groups at different concentrations, with an increase in the dependence of the toxic effect with increasing doses. The number of germ cell apoptosis was significantly (*p* < 0.001) increased for acute exposure to PM_2.5_ at concentrations of 10–1000 µg/mL. Exposure to concentrations above 100 µg/mL of PM_2.5_ significantly affected the degree of germ cell apoptosis in nematodes, implying that exposure to high concentrations of PM_2.5_ caused greater damage to nematode gonads. Exposure to 100–1000 µg/mL of PM_2.5_ in spring and winter induced higher numbers of germ cell apoptosis, and was highest in winter and lowest in summer of all four seasons (Figure 5a). Previous studies had shown that PM_2.5_ was more cytotoxic in winter than in summer, which was consistent with the results of this study [55]. We observed that the number of germ cell apoptosis was significantly (*p* < 0.05) increased by acute exposure to all concentrations of PM_1_ samples, and higher numbers of germ cell apoptosis were induced by exposure to 100–1000 µg/mL concentrations of PM_1_ in autumn and winter. It was noteworthy that the number of apoptosis caused by PM_1_ differed significantly between summer and winter, showing the highest number of apoptotic spots induced in winter and the lowest in summer (Figure 5b), which may be related to the high proportion of the organic fraction in winter. In comparison between the two particle sizes, low-particle-size PM_1_ caused a higher number of germ cell apoptosis than PM_2.5_. Thus, PM could cause gonad damage in nematodes, and exposure to high concentrations of PM_1_ in winter caused the greatest damage to nematodes’ gonads.

### 3.4. Correlation between PM Composition and Toxicity

**Figure 6:**
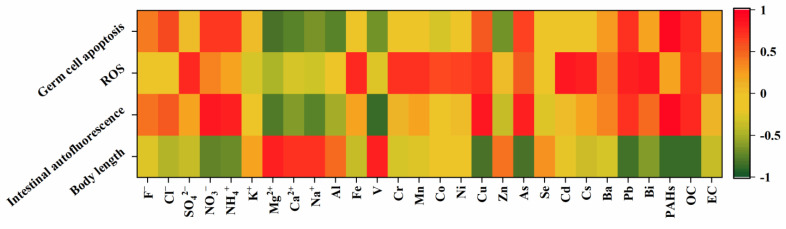
Heat map of correlation coefficients between body length/intestinal fluorescence/ROS/germ cell apoptosis and PM composition.

Previous studies had found that it was the chemical composition of fine particles, rather than their size, that determines the biotoxic effects caused by exposure [32]. The heat map of correlation coefficients between nematode toxicity indicators and components was shown (Figure 6). The highest correlation coefficients between body length and single chemical components were NO_3_^−^ (R = −0.741), NH_4_^+^ (R = −0.700), As (R = −0.818), Pb (R = −0.849), Cu (R = −0.809), PAHs (R = −0.871) and OC (R = −0.875). Intestinal autofluorescence was associated with inorganic ions such as NO_3_^−^ (R = 0.843), NH_4_^+^ (R = 0.792), some transition metal elements such as As (R = 0.802), Pb (R = 0.721) and Cu (R = 0.828) and organic fraction such as PAHs (R = 0.921) and OC (R = 0.756). Germ cell apoptosis had the highest correlation coefficients with Cl^−^ (R = 0.602), NO_3_^−^ (R = 0.676) and NH_4_^+^ (R = 0.669) among the water-soluble ions, As (R = 0.660) and Cu (R = 0.520) among the metal elements and PAHs (R = 0.925) and OC (R = 0.738) among the carbon-containing fractions. All three indicators were highly correlated with NO_3_^−^, NH_4_^+^, Cu, As, Pb, PAHs and OC, with the organic carbon component OC and PAHs showing the strongest correlation. The toxicity effects of these three indicators were the weakest in the summer PM samples and the strongest in the winter, which may be related to the enrichment of OC and PAHs in the winter samples. ROS was mainly associated with some transition metal elements, e.g., inorganic ions such as SO_4_^2−^ (R = 0.743), Fe (R = 0.754), Cr (R = 0.706), Mn (R = 0.698), Co (R = 0.627), Ni (R = 0.643), Cu (R = 0.724), Cd (R = 0.816), Cs (R = 0.805), Pb (R = 0.785), Bi (R = 0.815) and the carbon-containing component OC (R = 0.710) (Table S5), but did not correlate with PAHs. Thus, the above transition metal elements and carbon-containing component OC induced ROS production. This had been confirmed by previous studies where metals such as Pb, Cr, Ni, Fe and Cu induced ROS production in nematodes [25]. In addition, the secondary product of the photochemical reaction, SO_4_^2−^, also played a major role in the pro-inflammatory response and oxidative stress [45]. Organic extractable OEM_2.5−0.3_ significantly induced ROS overproduction and oxidative damage [56]. PAHs, due to their relatively small contribution to PM, may have their effects masked by other components. The toxic effects on nematodes were more relevant with organic fractions such as OC and PAHs; therefore, the organic fraction of PM was the key factor in the toxicity of PM to *C. elegans*.

### 3.5. Transgenerational Effects of PM_2.5_ and PM_1_ on C. elegans

**Figure 7:**
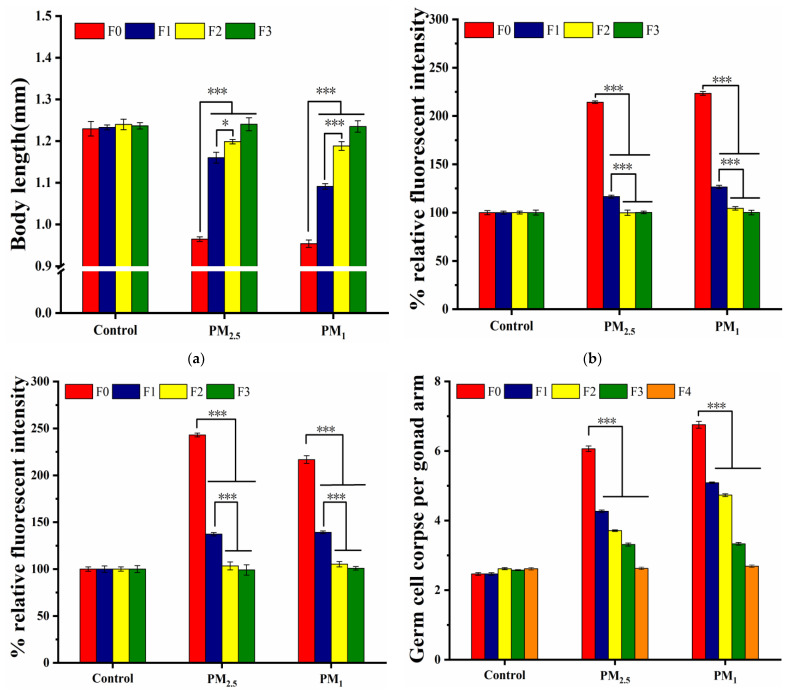
Effects of PM_2.5_ and PM_1_ exposure on nematodes and their offspring: (**a**) body length; (**b**) intensity of intestinal autofluorescence; (**c**) ROS production; (**d**) number of apoptotic germ cells. L4 stage larvae were selected for 24 h acute exposure. PM_2.5_ and PM_1_ samples in winter were selected and the exposure concentrations of PM were 1000 µg/mL. Bars represent means ± SEM. * *p* < 0.05, *** *p* < 0.001.

In this study, we investigated the cross-generational properties of nematode toxicity in response to exposure to PM_2.5_ and PM_1_. We observed that exposure to high concentrations of PM_2.5_ and PM_1_ adversely affected the growth, senescence, oxidative stress and germinal organs of exposed nematode progeny (Figure 7). Previous studies had shown that toxicity can be transmitted from exposed parent nematodes to their offspring [31].

Under maternal exposure, 1000 µg/mL of PM_2.5_ and PM_1_ significantly reduced the body length of nematodes in the F0 generation and gradually returned to control levels of body length in the F1–F3 generations. Exposure to the staining solution of PM_2.5_ and PM_1_ at high concentrations resulted in significant recovery in the F1 generation (*p* < 0.001). It was noteworthy that exposure to PM_1_ samples resulted in significantly lower recovery in F1 generation length than PM_2.5_. This was consistent with the results mentioned above, with lower particle sizes resulting in higher toxic effects. The body length of exposed nematodes returned to control levels in the F3 generation (Figure 7a). This proved that PM_2.5_ and PM_1_ significantly inhibit the growth and development of nematode progeny.

We observed that fine PM induced increased intestinal autofluorescence and ROS in nematode progeny (F1–F2) compared to controls, suggesting a multigenerational transmission of the effects of PM on intestinal damage in nematodes. The fluorescence intensities caused by high concentrations of PM_2.5_ and PM_1_ were significantly lower in the F1 generation compared to the F0 generation (*p* < 0.001). The recovery was highest in the F1 generation and returned to control levels in the F2-F3 generation, indicating marked recovery from intestinal damage (Figure 7b,c). The result was consistent with previous studies on the intergenerational effects of traffic- and coal-combustion-related PM_2.5_ on ROS in nematodes [27,29].

Exposure to high concentrations of fine PM induced a large number of apoptotic germ cells in the F0 generation of nematodes compared to the control, with significant differences between F0 and other generations (*p* < 0.001). We observed that exposure to PM_1_ induced significantly larger numbers of germ cells in the F1 and F2 generations than in PM_2.5_. Multigenerational reproductive toxicity induced by both particle sizes returned to the same level in the F3 generation and both returned to control levels in the F4 generation (Figure 7d). The number of offspring affected by maternal reproductive damage was larger compared to the multigenerational genotoxicity of nematode growth, Senescence level and oxidative stress levels. This suggested that gonadal damage to nematodes from exposure to PM was more pronounced and prolonged. Reproductive toxicity may, therefore, be a more sensitive and important endpoint for assessing cross-generational toxicity of fine PM [28].

## 4. Conclusions

In this study, toxicity in *C. elegans* was investigated by acute exposure to PM_2.5_ and PM_1_ collected from four seasons. Toxicological results showed that both PM_2.5_ and PM_1_ caused growth retardation, accelerated senescence, increased levels of oxidative stress and gonadal damage in a dose-dependent manner. In Lin’an, nematode toxicity when exposed to PM_2.5_ and PM_1_ in winter was higher than in summer, and the study found that this may be due to the fact that the organic fraction of PM is generally lower in summer than in winter. The exception was that exposure to PM_1_ samples in summer produced higher levels of ROS than in other seasons, which may be associated with the accumulation of certain transition metals, i.e., Fe, Cu, Cr, Mn, Co, Cd, Ni and Pb. Thus, OC, PAHs and certain transition metals played an important role in the toxicity of fine PM to nematodes. Overall, nematode toxicity induced by exposure to PM_1_ was significantly higher than that induced by PM_2.5_, suggesting that lower particle sizes induced greater nematode toxicity in vivo. This may be due to the relatively high proportion of OC and PAHs in PM_1_, and therefore the organic fraction was the key component of PM-induced biotoxicity.

In addition, this study showed that PM had an adverse effect on nematode offspring. Intestinal autofluorescence and ROS were substantially restored in the F1 generation, and both fluorescence indicators and body length were restored to control levels in the F3 generation. Germ cell apoptosis gradually returned to control levels in the F4 generation, suggesting that the adverse effects of PM_2.5_ and PM_1_ on gonadal damage were greater and less easily recovered. The evidence from this study suggests that parental exposure to fine PM may be toxic to maternal environmental organisms and even to humans and their offspring, with more profound reproductive effects.

## Figures and Tables

**Table 1:** Percentage of PAHs in PM_1_ and PM_2.5_ in four seasons.

PAHs	Spring		Summer		Autumn		Winter	
	PM_1_	PM_2.5_	PM_1_	PM_2.5_	PM_1_	PM_2.5_	PM_1_	PM_2.5_
Ratio	0.0140%	0.0101%	0.0041%	0.0036%	0.0121%	0.0079%	0.0200%	0.0142%

## Data Availability

Not applicable.

## Supplementary Materials

The following supporting information can be downloaded at: https://www.mdpi.com/article/10.3390/toxics11020116/s1, Table S1: Average concentrations of PM_2.5_ and PM_1_ in Lin’an; Table S2: The percentage of inorganic ions in PM_2.5_ and PM_1_ from four seasons; Table S3: The proportion of heavy metals in PM_2.5_ and PM_1_ from four seasons; Table S4: Average concentrations of OCEC in PM_2.5_ and PM_1_ from four seasons; Table S5: Correlation coefficients between the Body length/Intestinal fluorescence/ROS/Germ cell apoptosis and PM compositions; Table S6: The nomenclature table in the text.

## References

[1] Ostro B., Broadwin R., Green S., Feng W.Y., Lipsett M. (2006). Fine particulate air pollution and mortality in nine California counties: Results from CALFINE. Environ. Health Perspect..

[2] Landkocz Y., Ledoux F., Andre V., Cazier F., Genevray P., Dewaele D., Martin P.J., Lepers C., Verdin A., Courcot L. (2017). Fine and ultrafine atmospheric particulate matter at a multi-influenced urban site: Physicochemical characterization, mutagenicity and cytotoxicity. Environ. Pollut..

[3] Hu K., Guo Y., Hu D., Du R., Yang X., Zhong J., Fei F., Chen F., Chen G., Zhao Q. (2018). Mortality burden attributable to PM_1_ in Zhejiang province, China. Environ. Int..

[4] Yang B.Y., Guo Y., Morawska L., Bloom M.S., Markevych I., Heinrich J., Dharmage S.C., Knibbs L.D., Lin S., Yim S.H. (2019). Ambient PM_1_ air pollution and cardiovascular disease prevalence: Insights from the 33 Communities Chinese Health Study. Environ. Int..

[5] Yang B.Y., Guo Y., Bloom M.S., Xiao X., Qian Z.M., Liu E., Howard S.W., Zhao T., Wang S.Q., Li S. (2019). Ambient PM_1_ air pollution, blood pressure, and hypertension: Insights from the 33 Communities Chinese Health Study. Environ. Res..

[6] Wang X., Xu Z., Su H., Ho H.C., Song Y., Zheng H., Hossain M.Z., Khan M.A., Bogale D., Zhang H. (2021). Ambient particulate matter (PM_1_, PM_2.5_, PM_10_) and childhood pneumonia: The smaller particle, the greater short-term impact?. Sci. Total Environ..

[7] Yin G., Liu C., Hao L., Chen Y., Wang W., Huo J., Zhao Q., Zhang Y., Duan Y., Fu Q. (2019). Associations between size-fractionated particle number concentrations and COPD mortality in Shanghai, China. Atmos. Environ..

[8] Liu F., Guo Y., Liu Y., Chen G., Wang Y., Xue X., Liu S., Huo W., Mao Z., Hou Y. (2019). Associations of long-term exposure to PM_1_, PM_2.5_, NO_2_ with type 2 diabetes mellitus prevalence and fasting blood glucose levels in Chinese rural populations. Environ. Int..

[9] Wang J., Pan Y., Tian S., Chen X., Wang L., Wang Y. (2016). Size distributions and health risks of particulate trace elements in rural areas in northeastern China. Atmos. Res..

[10] Zhang H.H., Li Z., Liu Y., Xinag P., Cui X.Y., Ye H., Hu B.L., Lou L.P. (2018). Physical and chemical characteristics of PM_2.5_ and its toxicity to human bronchial cells BEAS-2B in the winter and summer. J. Zhejiang Univ. Sci. B.

[11] Velali E., Papachristou E., Pantazaki A., Choli-Papadopoulou T., Planou S., Kouras A., Manoli E., Besis A., Voutsa D., Samara C. (2016). Redox activity and in vitro bioactivity of the water-soluble fraction of urban particulate matter in relation to particle size and chemical composition. Environ. Pollut..

[12] Hassanvand M.S., Naddafi K., Faridi S., Arhami M., Nabizadeh R., Sowlat M.H., Pourpak Z., Rastkari N., Momeniha F., Kashani H. (2014). Indoor/outdoor relationships of PM_10_, PM_2.5_, and PM_1_ mass concentrations and their water-soluble ions in a retirement home and a school dormitory. Atmos. Environ..

[13] Widziewicz K., Rogula-Kozłowska W., Loska K. (2016). Cancer risk from arsenic and chromium species bound to PM_2.5_ and PM_1_—Polish case study. Atmos. Pollut. Res..

[14] Jiang N., Duan S., Yu X., Zhang R., Wang K. (2018). Comparative major components and health risks of toxic elements and polycyclic aromatic hydrocarbons of PM_2.5_ in winter and summer in Zhengzhou: Based on three-year data. Atmos. Res..

[15] Lin H., Tao J., Du Y., Liu T., Qian Z., Tian L., Di Q., Rutherford S., Guo L., Zeng W. (2016). Particle size and chemical constituents of ambient particulate pollution associated with cardiovascular mortality in Guangzhou, China. Environ. Pollut..

[16] Niu X., Ho S.S.H., Ho K.F., Huang Y., Sun J., Wang Q., Zhou Y., Zhao Z., Cao J. (2017). Atmospheric levels and cytotoxicity of polycyclic aromatic hydrocarbons and oxygenated-PAHs in PM_2.5_ in the Beijing-Tianjin-Hebei region. Environ. Pollut..

[17] Totlandsdal A.I., Lag M., Lilleaas E., Cassee F., Schwarze P. (2015). Differential proinflammatory responses induced by diesel exhaust particles with contrasting PAH and metal content. Environ. Toxicol..

[18] Kamal A., Qamar K., Gulfraz M., Anwar M.A., Malik R.N. (2015). PAH exposure and oxidative stress indicators of human cohorts exposed to traffic pollution in Lahore city (Pakistan). Chemosphere.

[19] Qi Z., Zhang Y., Chen Z.F., Yang C., Song Y., Liao X., Li W., Tsang S.Y., Liu G., Cai Z. (2020). Chemical identity and cardiovascular toxicity of hydrophobic organic components in PM_2.5_. Ecotoxicol. Environ. Saf..

[20] Niu X., Wang Y., Ho S.S.H., Chuang H.C., Sun J., Qu L., Wang G., Ho K.F. (2021). Characterization of organic aerosols in PM_1_ and their cytotoxicity in an urban roadside area in Hong Kong. Chemosphere.

[21] Kaletta T., Hengartner M.O. (2006). Finding function in novel targets: *C. elegans* as a model organism. Nat. Rev. Drug Discov..

[22] Leung M.C., Williams P.L., Benedetto A., Au C., Helmcke K.J., Aschner M., Meyer J.N. (2008). *Caenorhabditis elegans*: An emerging model in biomedical and environmental toxicology. Toxicol. Sci..

[23] Hoss S., Jansch S., Moser T., Junker T., Rombke J. (2009). Assessing the toxicity of contaminated soils using the nematode *Caenorhabditis elegans* as test organism. Ecotoxicol. Environ. Saf..

[24] Zhou D., Yang J., Li H., Cui C., Yu Y., Liu Y., Lin K. (2016). The chronic toxicity of bisphenol A to *Caenorhabditis elegans* after long-term exposure at environmentally relevant concentrations. Chemosphere.

[25] Sun L., Wu Q., Liao K., Yu P., Cui Q., Rui Q., Wang D. (2016). Contribution of heavy metals to toxicity of coal combustion related fine particulate matter (PM_2.5_) in *Caenorhabditis elegans* with wild-type or susceptible genetic background. Chemosphere.

[26] Liu H., Tian L., Wang S., Wang D. (2021). Size-dependent transgenerational toxicity induced by nanoplastics in nematode *Caenorhabditis elegans*. Sci. Total Environ..

[27] Zhao Y., Lin Z., Jia R., Li G., Xi Z., Wang D. (2014). Transgenerational effects of traffic-related fine particulate matter (PM_2.5_) on nematode *Caenorhabditis elegans*. J. Hazard Mater..

[28] Sun L., Lin Z., Liao K., Xi Z., Wang D. (2015). Adverse effects of coal combustion related fine particulate matter (PM_2.5_) on nematode *Caenorhabditis elegans*. Sci. Total Environ..

[29] Wang M., Nie Y., Liu Y., Dai H., Wang J., Si B., Yang Z., Cheng L., Liu Y., Chen S. (2019). Transgenerational effects of diesel particulate matter on *Caenorhabditis elegans* through maternal and multigenerational exposure. Ecotoxicol. Environ. Saf..

[30] Wang D.-y., Yang P. (2007). Multi-biological defects caused by lead exposure exhibit transferable properties from exposed parents to their progeny in *Caenorhabditis elegans*. J. Environ. Sci..

[31] Wang D., Shen L., Wang Y. (2007). The phenotypic and behavioral defects can be transferred from zinc-exposed nematodes to their progeny. Environ. Toxicol. Pharmacol..

[32] Longhin E., Pezzolato E., Mantecca P., Holme J.A., Franzetti A., Camatini M., Gualtieri M. (2013). Season linked responses to fine and quasi-ultrafine Milan PM in cultured cells. Toxicol. Vitr..

[33] Nie D., Wu Y., Chen M., Liu H., Zhang K., Ge P., Yuan Y., Ge X. (2018). Bioaccessibility and health risk of trace elements in fine particulate matter in different simulated body fluids. Atmos. Environ..

[34] Cao J.J., Lee S.C., Ho K.F., Zhang X.Y., Zou S.C., Fung K., Chow J.C., Watson J.G. (2002). Characteristics of carbonaceous aerosol in Pearl River Delta Region, China during 2001 winter period. Atmos. Environ..

[35] Brenner S. (1974). The genetics of *Caenorhabditis elegans*. Genetics.

[36] Williams P.L., Dusenbery D.B. (1990). A promising indicator of neurobehavioral toxicity using the nematode *Caenorhabditis elegans* and computer tracking. Toxicol. Ind. Health.

[37] Shen L., Hu Y., Cai T., Lin X., Wang D. (2010). Regulation of longevity by genes required for the functions of AIY interneuron in nematode *Caenorhabditis elegans*. Mech. Ageing Dev..

[38] Wang X., Shen L., Yu H., Wang D. (2008). Toxicity evaluation in a paper recycling mill effluent by coupling bioindicator of aging with the toxicity identification evaluation method in nematode *Caenorhabditis elegans*. Environ. Sci..

[39] Zhao Y., Jin L., Wang Y., Kong Y., Wang D. (2019). Prolonged exposure to multi-walled carbon nanotubes dysregulates intestinal mir-35 and its direct target MAB-3 in nematode *Caenorhabditis elegans*. Sci. Rep..

[40] Yang Y., Dong W., Wu Q., Wang D. (2021). Response of G protein-coupled receptor CED-1 in germline to polystyrene nanoparticles in *Caenorhabditis elegans*. Nanoscale Adv..

[41] Kelly K.O., Am S.G.V., Dernburg A.F. (2000). *Caenorhabditis elegans* msh-5 is required for both normal and radiation-induced meiotic crossing over but not for completion of meiosis. Genetics.

[42] Shi M., Wu H., Zhang S., Li H., Yang T., Liu W., Liu H. (2014). Weekly cycle of magnetic characteristics of the daily PM_2.5_ and PM_2.5–10_ in Beijing, China. Atmos. Environ..

[43] Meng C.C., Wang L.T., Zhang F.F., Wei Z., Ma S.M., Ma X., Yang J. (2016). Characteristics of concentrations and water-soluble inorganic ions in PM_2.5_ in Handan City, Hebei province, China. Atmos. Res..

[44] Zhang T., Cao J.J., Tie X.X., Shen Z.X., Liu S.X., Ding H., Han Y.M., Wang G.H., Ho K.F., Qiang J. (2011). Water-soluble ions in atmospheric aerosols measured in Xi’an, China: Seasonal variations and sources. Atmos. Res..

[45] Perrone M.G., Gualtieri M., Ferrero L., Lo Porto C., Udisti R., Bolzacchini E., Camatini M. (2010). Seasonal variations in chemical composition and in vitro biological effects of fine PM from Milan. Chemosphere.

[46] Lai S., Zhao Y., Ding A., Zhang Y., Song T., Zheng J., Ho K.F., Lee S.C., Zhong L. (2015). Characterization of PM_2.5_ and the major chemical components during a 1-year campaign in rural Guangzhou, Southern China. Atmos. Res..

[47] Arimoto R., Duce R.A., Savoie D.L., Prospero J.M., Talbot R., Cullen J.D., Tomza U., Lewis N.F., Ray B.J. (1996). Relationships among aerosol constituents from Asia and the North Pacific during PEM-West A. J. Geophys. Res. Atmos..

[48] Di A., Wu Y., Chen M., Nie D., Ge X. (2020). Chemical Characterization of Seasonal PM_2.5_ Samples and Their Cytotoxicity in Human Lung Epithelial Cells (A549). Int. J. Environ. Res. Public Health.

[49] Manzano-Leon N., Serrano-Lomelin J., Sanchez B.N., Quintana-Belmares R., Vega E., Vazquez-Lopez I., Rojas-Bracho L., Lopez-Villegas M.T., Vadillo-Ortega F., De Vizcaya-Ruiz A. (2016). TNFα and IL-6 Responses to Particulate Matter in Vitro: Variation According to PM Size, Season, and Polycyc.clic Aromatic Hydrocarbon and Soil Content. Environ. Health Perspect..

[50] Teixeira E.C., Agudelo-Castañeda D.M., Fachel J.M.G., Leal K.A., Garcia K.d.O., Wiegand F. (2012). Source identification and seasonal variation of polycyclic aromatic hydrocarbons associated with atmospheric fine and coarse particles in the Metropolitan Area of Porto Alegre, RS, Brazil. Atmos. Res..

[51] Zhang Y., Zhang H., Deng J., Du W., Hong Y., Xu L., Qiu Y., Hong Z., Wu X., Ma Q. (2017). Source regions and transport pathways of PM_2.5_ at a regional background site in East China. Atmos. Environ..

[52] Zhang R., Jing J., Tao J., Hsu S.C., Wang G., Cao J., Lee C.S.L., Zhu L., Chen Z., Zhao Y. (2013). Chemical characterization and source apportionment of PM_2.5_ in Beijing: Seasonal perspective. Atmos. Chem. Phys..

[53] Wu Q., Yin L., Li X., Tang M., Zhang T., Wang D. (2013). Contributions of altered permeability of intestinal barrier and defecation behavior to toxicity formation from graphene oxide in nematode *Caenorhabditis elegans*. Nanoscale.

[54] Quijano M.F.C., de Paula Ribeiro J., Josende M.E., Santa-Helena E., De Falco A., Gioda C.R., Gioda A. (2022). Assessment of the effects of seasonality on the ecotoxicity induced by the particulate matter using the animal model *Caenorhabditis elegans*. Chemosphere.

[55] Chen Y., Luo X.S., Zhao Z., Chen Q., Wu D., Sun X., Wu L., Jin L. (2018). Summer-winter differences of PM_2.5_ toxicity to human alveolar epithelial cells (A549) and the roles of transition metals. Ecotoxicol. Environ. Saf..

[56] Badran G., Verdin A., Grare C., Abbas I., Achour D., Ledoux F., Roumie M., Cazier F., Courcot D., Lo Guidice J.M. (2020). Toxicological appraisal of the chemical fractions of ambient fine (PM_2.5-0.3_) and quasi-ultrafine (PM_0.3_) particles in human bronchial epithelial BEAS-2B cells. Environ. Pollut..

