# Supplementary material for: Chemical Composition and Transgenerational Effects on Caenorhabditis elegans of Seasonal Fine Particulate Matter
Source: Toxics. 2023 Jan 24;11(2):116. doi: 10.3390/toxics11020116 (PMC9964627; doi:10.3390/toxics11020116)
Supplement: Supplementary file 1 [file toxics-11-00116-s001.zip › toxics-2133860-supplementary.pdf]

Table S1. Average concentrations of $PM_{2.5}$ and PM_1 in Lin'an.

	Average concentrations of $PM_{2.5}$($\mu g.m^{-3}$)	Average concentrations of PM_1($\mu g.m^{-3}$)
Spring	42.53±15.56	32.92±8.94
Summer	25.68±6.82	20.70±5.92
Autumn	38.16±14.97	29.22±12.43
Winter	58.69±23.67	43.56±16.31

Table S2. The percentage of inorganic ions in $PM_{2.5}$ and PM_1 from four seasons.

Inorganic ions	Spring		Summer		Autumn		Winter	
	$PM_{2.5}$	PM_1	$PM_{2.5}$	PM_1	$PM_{2.5}$	PM_1	$PM_{2.5}$	PM_1
F^-	0.20%	0.16%	0.08%	0.13%	0.15%	0.15%	0.10%	0.17%
Cl^-	0.78%	0.61%	0.41%	0.42%	0.79%	0.79%	0.70%	0.75%
SO_4^{2-}	16.51%	12.95%	15.23%	16.10%	14.89%	15.44%	17.21%	16.00%
NO_3^-	17.84%	13.99%	6.13%	5.22%	8.40%	9.96%	19.45%	19.19%
NH_4^+	8.57%	6.92%	5.27%	4.48%	6.33%	6.57%	8.67%	8.95%
K^+	1.13%	0.86%	0.77%	0.79%	0.99%	1.03%	0.77%	0.80%
Mg^{2+}	0.44%	0.33%	0.51%	0.39%	0.44%	0.35%	0.32%	0.36%
Ca^{2+}	1.31%	0.65%	1.20%	0.81%	1.01%	0.64%	0.72%	0.61%
Na^+	2.58%	2.85%	3.28%	3.65%	2.92%	2.77%	1.62%	2.25%
SNA	42.92%	33.86%	26.63%	25.79%	29.62%	31.97%	45.33%	44.14%
Ratio	49.36%	39.31%	32.88%	31.99%	35.92%	37.69%	49.57%	49.07%
NO_3^-/ SO_4^{2-}	1.08	1.08	0.40	0.32	0.56	0.65	1.13	1.20

Table S3. The proportion of heavy metals in $PM_{2.5}$ and PM_1 from four seasons(μg/g).

Metals	Spring		Summer		Autumn		Winter	
	$PM_{2.5}$	PM_1	$PM_{2.5}$	PM_1	$PM_{2.5}$	PM_1	$PM_{2.5}$	PM_1
Al	5362.7±122.1	4251.3±107.3	5926.6±157.5	6111.9±172.2	6347.3±189.0	4675.0±117.4	5340.1±134.2	5076.9±136.5
Fe	12567.4±322.4	10854.3±225.6	12082.2±276.2	15582.1±387.2	13602.8±341.8	12800.2±176.5	16411.2±189.1	13211.5±268.8
V	28.0±0.7	24.0±2.0	29.6±2.5	30.7±6.9	28.4±9.6	30.3±5.1	21.8±4.5	16.0±2.0
Cr	166.7±2.8	137.8±1.9	175.4±2.1	186.0±2.7	162.7±1.8	153.8±4.5	194.0±2.7	179.9±3.8
Mn	163.8±1.0	143.9±0.2	161.9±0.3	170.1±0.7	153.2±0.7	141.0±0.4	193.8±0.5	158.9±0.3
Co	17.3±0.8	14.0±1.4	17.0±1.7	19.5±2.5	17.7±3.0	16.8±2.1	18.8±1.8	17.0±1.7
Ni	123.5±19.5	103.3±5.8	134.2±7.1	136.8±9.9	121.8±14.3	115.9±13.4	143.6±12.6	132.0±7.6
Cu	304.5±8.0	291.8±5.1	258.5±3.9	296.2±0.3	276±10.7	271.3±2.5	355.3±9.0	326.2±1.7
Zn	4045.3±100.8	3031.3±31.5	3894.2±148.7	3430.4±84.8	3675.6±141.4	2891.3±82.3	3946.5±84.5	2940.9±66.2
As	105.4±3.4	98.6±6.9	85.4±4.9	106.6±8.1	105.0±17.3	104.4±9.9	107.3±12.0	115.6±0.7
Se	55.8±5.4	46.2±4.5	40.5±5.8	50.4±6.2	55.8±5.5	57.9±9.1	44.6±2.7	38.4±6.1
Cd	47.8±0.3	43.8±0.3	47.9±0.3	59.1±0.8	46.6±1.8	46.3±1.6	53.7±0.6	49.5±0.4
Cs	20.2±0.2	19.1±0.2	20.8±0.5	25.9±0.7	21.1±0.8	20.6±0.4	24.6±0.8	22.4±0.3
Ba	18.4±0.1	15.6±0.2	12.6±0.2	14.6±0.4	16.1±0.5	16.5±0.2	21.2±0.4	13.4±0.3
Pb	234.2±10.4	206.8±3.1	180.8±1.3	248.3±3.4	216.6±6.0	255.5±1.3	268.2±8.0	261.3±3.0
Bi	53.5±0.5	49.2±1.1	53.1±0.9	66.5±1.3	56.3±2.9	54.3±1.4	75.5±3.6	64.6±1.2
Ratio	2.3%	1.9%	2.3%	2.7%	2.5%	2.2%	2.7%	2.3%

Table S4. Average concentrations of OCEC in $PM_{2.5}$ and PM_1 from four seasons($\mu g/m^3$).

	Spring		Summer		Autumn		Winter	
	**$PM_{2.5}$**	**PM_1**	**$PM_{2.5}$**	**PM_1**	**$PM_{2.5}$**	**PM_1**	**$PM_{2.5}$**	**PM_1**
OC	8.32±1.57	6.83±0.88	4.75±2.26	4.45±2.16	7.26±1.38	6.11±2.05	12.60±1.39	9.79±0.35
EC	0.66±0.19	0.56±0.08	0.53±0.07	0.50±0.13	0.75±0.11	0.67±0.18	1.20±0.20	1.03±0.15
TC	8.99±1.72	7.39±0.90	5.47±2.30	4.95±2.27	7.96±1.96	6.76±2.21	13.79±1.50	10.82±0.23
OC/EC	13.02	12.20	9.24	8.67	10.39	9.50	10.71	9.68
SOC	2.16	1.33	1.58	1.07	1.81	1.62	2.30	2.02
OM/PM	31.31%	33.17%	30.79%	34.36%	30.43%	33.47%	34.34%	35.95%

Table S5. Correlation coefficients between the Body length/ Intestinal fluorescence / ROS/Germ cell apoptosis and PM compositions.

Compositions	Body length	Fluorescence	ROS	Apoptosis
F-	-0.271	0.421	-0.055	0.388
Cl-	-0.460	0.534	-0.116	0.602
SO_4^{2-}	-0.366	0.201	0.743*	0.064
NO_3^-	-0.741*	0.843**	0.341	0.676
NH_4^+	-0.700	0.792*	0.232	0.669
K^+	0.191	-0.093	-0.313	0.052
Mg^{2+}	0.801	-0.803	-0.475	-0.813
Ca^{2+}	0.720	-0.642	-0.314	-0.770
Na^+	0.719	-0.749	-0.324	-0.649
Al	0.464	-0.528	-0.021	-0.752
Fe	-0.367	0.220	0.754*	-0.004
V	0.802	-0.857	-0.245	-0.672
Cr	-0.284	0.114	0.706	-0.137
Mn	-0.263	0.202	0.698	-0.143
Co	-0.084	-0.095	0.627	-0.276
Ni	-0.195	0.015	0.643	-0.207
Cu	-0.809*	0.828*	0.724*	0.520
Zn	0.432	-0.378	0.062	-0.662
As	-0.818*	0.802*	0.522	0.660
Se	0.284	-0.255	-0.160	-0.102
Cd	-0.219	0.061	0.816*	-0.173
Cs	-0.349	0.179	0.805*	-0.047
Ba	-0.315	0.375	0.390	0.244
Pb	-0.849**	0.721*	0.785*	0.696
Bi	-0.613	0.475	0.815*	0.229
PAHs	-0.871**	0.921**	0.192	0.925**
OC	-0.875**	0.756*	0.716*	0.738*
EC	-0.386	0.115	0.496	0.238

The correlation between two variables was considered significant if $p<0.05$ and statistically significant if $p<0.01$.

Table S6. The nomenclature table in the text.

Abbreviations	Full name
AO	acridine orange
BaA	Benzo(a)anthracene
BaP	Benzo(a)pyrene
BbF	Benzo(b)fluoranthene
C. elegans	Caenorhabditis elegans
CM-H2DCFDA	5',6'-chloromethyl-2',7'-dichlorodihydrofluorescein diacetate
CGC	Caenorhabditis Genetics Center
CVD	cardiovascular diseases
DaA	Dibenzo-(a,h)anthracene
EC	Elemental Carbon
GC-MS/MS	Gas chromatography-mass spectrometer
IC	Ion Chromatography
ICP-MS	Inductively Coupled Plasma Optical Emission Spectrometer
NGM	Nematode Growth Medium
OC	Organic Carbon
OM	Organic matter
POC	Primary Organic Carbon
PAHs	Polycyclic aromatic hydrocarbons
PM	particulate matter
ROS	reactive oxygen species
SOC	Second Organic Carbon
